# An ultra-sensitive biosensor based on surface plasmon resonance and weak value amplification

**DOI:** 10.3389/fchem.2024.1382251

**Published:** 2024-03-08

**Authors:** Lizhong Zhang, Mingyi He, Yang Xu, Cuixia Guo, Chongqi Zhou, Tian Guan

**Affiliations:** ^1^ Institute of Optical Imaging and Sensing, Shenzhen Key Laboratory for Minimal Invasive Medical Technologies, Shenzhen International Graduate School, Tsinghua University, Shenzhen, China; ^2^ School of International Education, Beijing University of Chemical Technology, Beijing, China; ^3^ School of Mechanical Engineering and Automation, Fuzhou University, Fuzhou, Fujian Province, China; ^4^ Department of Physics, Tsinghua University, Beijing, China

**Keywords:** weak value amplification, surface plasmon resonance, refractive index sensor, biosensor, biomolecular interaction

## Abstract

An ultra-sensitive phase plasmonic sensor combined with weak value amplification is proposed for the detection of IgG, as a model analyte. Phase detection is accomplished by self-interference between the p-polarization and the s-polarization of the light. With the principles of weak value amplification, a phase compensator is used to modulate the coupling strength and enhance the refractive index sensitivity of the system. On a simple Au-coated prism-coupled surface plasmon resonance (SPR) structure, the scheme, called WMSPR, achieves a refractive index sensitivity of 4.737 × 10^4^ nm/RIU, which is about three times higher than that of the conventional phase-based approach. The proposed WMSPR biosensor gives great characteristics with a high resolution of 6.333 × 10^−8^ RIU and a low limit of detection (LOD) of 5.3 ng/mL. The results yield a great scope to promote the optimization of other SPR biosensors for high sensitivity.

## 1 Introduction

Surface plasmon resonance (SPR) is widely used as a refractive index sensor in biomedical detection with the advantages of label-free and real-time sensing capabilities (Nelson et al., 2001; Wu et al., 2004; Beseničar et al., 2006; Dudak and Boyacı, 2009). Due to the enhanced interaction between the incident light and the surface plasmon, SPR sensors can obtain higher sensitivity and detection capability than other optical sensors. As the sensor’s sensitivity is one of the main parameters for sensor design, many works have proposed different structures of SPR sensors to improve the system’s sensitivity (Xu et al., 2019). In 2001, Neenniger et al. proposed a long-range SPR sensor with an additional layer of Teflon dielectric film between the Au film and the prism (Nenninger et al., 2001). This system has achieved a refractive index sensitivity of 5.7 × 10^4^ nm/RIU. In 2021, Satyendra et al. achieved a refractive index sensitivity of 1.03 × 10^4^ nm/RIU by coating the fiber surface with indium tin oxide and a polymer layer (Mishra and Mishra, 2021). Recently, many works have used nanomaterials to enhance the sensitivity by increasing the surface-to-volume ratio, such as graphene, transition metal dichalcogenides, and hexagonal boron nitride (Szunerits et al., 2013; Lin et al., 2016; Zhou et al., 2020; Divya et al., 2022). The sensitivities of these sensors are highly dependent on the material and structure of the sensors, and it is difficult to further optimize the existing sensor structure. On the other hand, these sophisticated nanomaterials are not convenient for the fabrication and application of the sensor. Therefore, it is necessary to propose a simple and universal method to enhance the sensitivity of SPR sensors. So far, several types of SPR techniques have been developed, like intensity (Prabowo et al., 2018), angular (Jang et al., 2015), wavelength (Homola et al., 2002), and phase (Shao et al., 2013) interrogation. For intensity, angular, and wavelength interrogation SPR sensors, the energy of the incident light is strongly coupled to the surface plasmon. In this case, the reflection minimum represents the resonance angle or resonance wavelength, which is called SPR dip. SPR dip position is related to the refractive index of the dielectric medium, which forms the basic principle of refractive index sensors. Meanwhile, the phase of p-polarized light also changes with the interaction of the incident light with the surface plasmon, while the s-polarized light remains unchanged. Therefore, a phase difference is generated between p, s polarized light, which introduces the principle of the phase interrogation SPR sensor. In the phase interrogation SPR sensor, the energy coupling between the incident light and the surface plasmon is weak, which can be applied to weak measurement systems.

Weak measurement (WM) and weak value amplification (WVA) have a wide range of applications for high-precision measurements (Alves et al., 2015; Zhang et al., 2015; Magaña-Loaiza et al., 2016). By introducing post-selection, the weak value amplification technique can improve the sensitivity of the weakly coupled system by more than two orders of magnitude. In 2008, Hosten et al. used weak value amplification to directly observe lateral shift between polarized light caused by the photon spin Hall effect (Hosten and Kwiat, 2008). With weak value amplification, they have observed a displacement of 1Å using a charge-coupled device (CCD), which is far below the resolution capability of CCD. Since then, the weak value amplification has been applied to various techniques, such as interferometer (Fang et al., 2018), fiber Bragg grating sensor (Salazar-Serrano et al., 2015), and ellipsometer (Zhou et al., 2023). Among them, the refractive index sensitivity of the ellipsometer-based weak measurement system is improved to 1.36 × 10^4^ nm/RIU, and its refractive index resolution is 4 × 10^−7^ RIU, which is close to the performance of the conventional SPR sensor (Ma et al., 2019). Luo and Xu et al. have combined the weak value amplification technique with the SPR sensor for the refractive index sensor (Luo et al., 2017; Xu et al., 2020). This scheme is optimized to obtain a higher optical intensity contrast with a refractive index resolution of 2.9 × 10^−7^ RIU. Although weak value amplification techniques have been applied to SPR sensors, systems to enhance the sensitivity of sensors have not been proposed.

In this work, we propose an approach that combines the phase interrogation SPR sensing principle with the weak value amplification technique, which is called a WMSPR system. WMSPR system uses a phase compensator to modulate the coupling strength and a post-selection process to enhance the sensitivity. The sensitivity of the system increases as the coupling strength decreases. For a simple Au-coated prism-coupled SPR, the WMSPR system achieves an ultra-high refractive index sensitivity of 4.737 × 10^4^ nm/RIU. Compared with the conventional phase interrogation SPR system, the sensitivity is amplified by a factor of about 3. In the WMSPR system, we achieve a refractive index resolution of 6.333 × 10^−8^ RIU. We used this system to detect IgG and Anti-IgG binding interaction as a demonstration experiment. The principles of the WMSPR system can be applied to various SPR sensors.

## 2 Materials and methods

### 2.1 Materials

Phosphate buffered solution (PBS, powder) is purchased from Solarbio Science & Technology Company (Beijing, China). The goat anti-rabbit IgG and rabbit IgG are purchased from Bioss (Beijing, China). Dopamine (Aladdin, Shanghai, China) is dissolved in Tris (Aladdin, Shanghai, China) solution at pH 8.5 to prepare 0.01 g/L of dopamine solution. 2% no protein blocking solution is purchased from Sangon Biotech, Shanghai, China.

### 2.2 Methods

**FIGURE 1:**
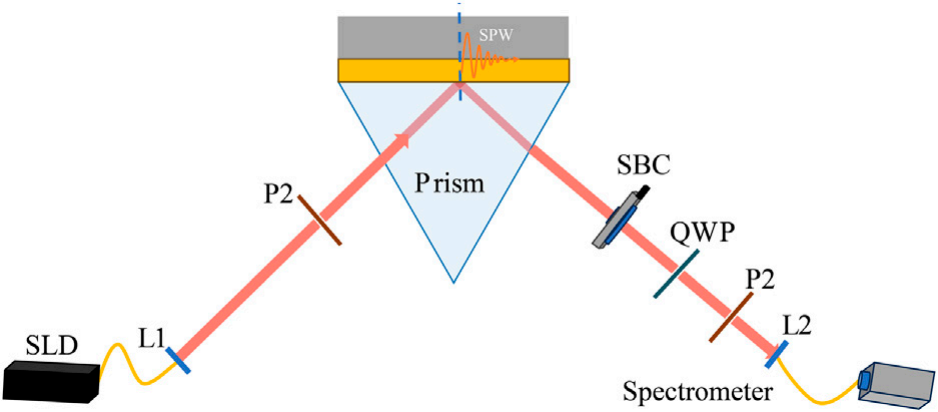
Scheme of the WMSPR sensor. Light source: superluminescent diodes (SLD); L1 and L2, achromatic lens; P1 and P2, Polarizer; SBC, Soleil-Babinet compensator.

In Figure 1, we depict the WMSPR system of combining phase interrogation with weak value amplification. Here, we adopt the Kretschmann configuration as an example to illustrate the principles of our scheme. It consists of a ZF6 prism with high refractive index *n*_p_ = 1.75, a thin Au layer with relative permittivity *ɛ*_m_, and a dielectric layer with low refractive index *n*_d_ = 1.333. To excite SPPs that propagate along the metal-dielectric interface, the phase-matching condition needs to be fulfilled

$$k_{0}n_{\text{p}}sin\theta =Re\left\{k_{0}\sqrt{\frac{\varepsilon_{\text{m}}n_{\text{d}}^{2}}{\varepsilon_{\text{m}}+n_{\text{d}}^{2}}}\right\},$$ (1)

where *θ* is the incident angle. The light source is a broadband superluminescent diode (SLD) which is centered at 830 nm. According to Eq. 1, the SPPs would be excited at the incident angle of *θ* = 53.4°. By applying the Fresnel theory, we can obtain the reflection coefficient *r*_*p*(*s*)_ for the p and s polarized incident light as follows (Kihm et al., 2012)

$$r_{p\left(s\right)}=\frac{r_{31}^{p\left(s\right)}+r_{21}^{p\left(s\right)}exp\left(ik_{z1}d\right)}{1+r_{31}^{p\left(s\right)}r_{21}^{p\left(s\right)}exp\left(ik_{z1}d\right)}$$ (2)

in which *d* is the thickness of the metal layer. In Eq. 2, we label the metal layer, prism, and dielectric medium with 1, 2, and 3.

In our scheme, the light beam is first selected in the linear polarization state

$$|\Psi_{i}\rangle =cos\alpha |p\rangle +sin\alpha |s\rangle ,$$ (3)

where |*p*⟩ and |*s*⟩ represent the TM mode (p-polarization) and TE (s-polarization) polarized light, respectively. The angle between the polarizer’s polarization axis and horizontal direction is *α*.

**FIGURE 2:**
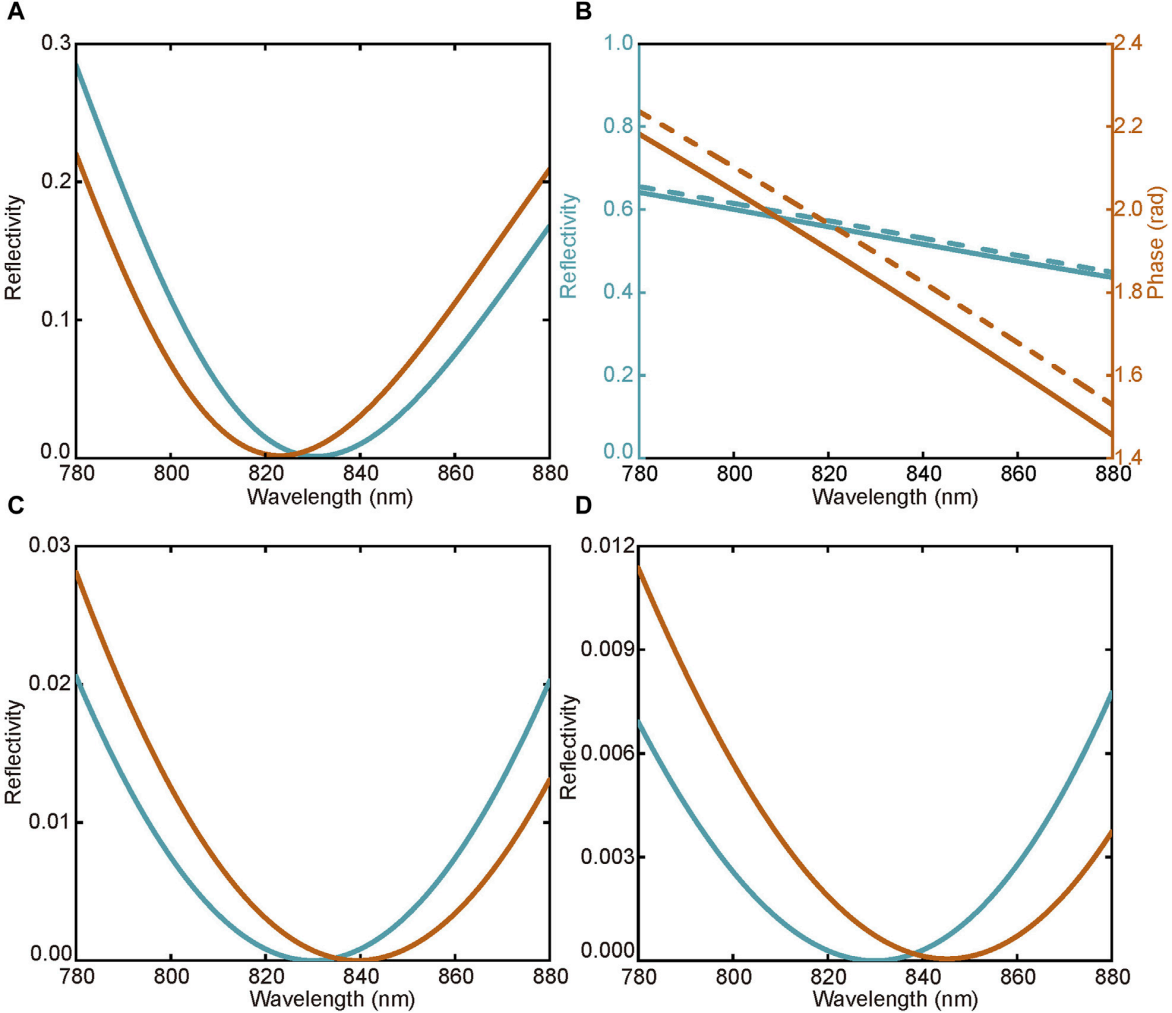
Simulational results. **(A)** Reflectivity curves for wavelength interrogation SPR. The red and blue curves represent the conditions of *n*_*d*_ = 1.333 and *n*_*d*_ = 1.334, respectively. **(B)** The reflectivity and phase for Au-coated SPR when d = 25 nm. **(C)** Reflectivity curves for phase interrogation SPR. **(D)** Reflectivity curves for WMSPR.

As SPPs can be only excited by p-polarized light, p and s-polarized light are endowed with different reflection coefficient

$$\begin{array}{ll} |\psi^{\prime }\rangle & =r_{p}cos\alpha |p\rangle +r_{s}sin\alpha |s\rangle \\ & =|r_{p}|e^{i\varphi_{p}\left(\lambda ,n_{\text{d}}\right)}cos\alpha |p\rangle +|r_{s}|e^{i\varphi_{s}\left(\lambda ,n_{\text{d}}\right)}sin\alpha |s\rangle , \end{array}$$ (4)

where |*r*_*p*(*s*)_|, *φ*_*p*(*s*)_ (*λ*, *n*_d_) reveal the amplitude and phase of reflection coefficient *r*_*p*(*s*)_, respectively. The value of *r*_*p*(*s*)_ is affected by the refractive index *n*_d_ of the dielectric layer. Conventional SPR mainly focuses on the amplitude shift, as shown in Figure 2A. When d = 45 nm, the p-polarized incident light is strongly coupled with the surface plasmon and causes an SPR dip in the reflectivity curve, which can be observed for wavelength or angular interrogation SPR sensor. Here, we focus on the variation of *φ*_*p*_ (*λ*, *n*_d_). As shown in Figure 2B, when d = 25 nm is far from the optimal resonance thickness, the SPR is quite weak, and the reflectance curves are flat. At this time, the phase rather than the amplitude varies significantly with the refractive index of the dielectric medium. Therefore, as shown in Figure 2B, there is a linear relationship between phase and wavelength. We make the approximations: *φ*_*p*_ (*λ*, *n*_d_) = *k*_1_ *λ* + *k*_2_ (*n*_d_). And |*r*_*p*_| and *k*_1_ are constants, *k*_2_ (*n*_d_) varies with *n*_d_. The reflectivity of s-polarization remains unchanged: *φ*_*s*_ (*λ*, *n*_d_) = *φ*_*s*_. Hence, Eq. 4 can be written as

$$|\psi^{\prime }\rangle =|r_{p}|e^{i\left(k_{1}\lambda +k_{2}\left(n_{\text{d}}\right)\right)}cos\alpha |p\rangle +|r_{s}|e^{i\varphi_{s}}sin\alpha |s\rangle .$$ (5)

After that, a Soleil-Babinet compensator (SBC) introduces an additional phase difference *φ* ≃ *lλ* between the *p* and *s* polarizations. After that, the system is involved in

$$|\psi^{\prime }\rangle =|r_{p}|e^{i\left[\left(k_{1}-1/2\right)\lambda +k_{2}\left(n_{\text{d}}\right)\right]}cos\alpha |p\rangle +|r_{s}|e^{i\left(\varphi_{s}+\lambda /2\right)}sin\alpha |s\rangle .$$ (6)

By adding a quarter-wave plate (QWP) after the SBC, we can convert the p-polarized light and s-polarized light into right-handed circularly polarized light $|+\rangle =\left(|p\rangle +i|s\rangle \right)/\sqrt{2}$ and left-handed circularly polarized light $|-\rangle =\left(|p\rangle -i|s\rangle \right)/\sqrt{2}$ , respectively. The angle between the fast axis direction of QWP and the horizontal direction is *π*/4. Hence, the system state can be expressed as

$$\begin{array}{cc} |\psi^{\prime }\rangle & =|r_{p}|e^{i\left[\left(k_{1}-1/2\right)\lambda +k_{2}\left(n_{\text{d}}\right)\right]}cos\alpha |p\rangle +|r_{s}|e^{i\left(\varphi_{s}+\lambda /2\right)}sin\alpha |s\rangle \\ & =e^{iK\left(\lambda ,n_{\text{d}}\right)\hat{A}}\left[|r_{p}|cos\alpha |+\rangle +|r_{s}|sin\alpha |-\rangle \right], \end{array}$$ (7)

where $\hat{A}=|+\rangle \langle +|-|-\rangle \langle -|$ is the system operator, and *K* (*λ*, *n*_d_) = [(*k*_1_ − *l*)*λ* + *k*_2_ (*n*_d_) − *φ*_*s*_]/2. We have $\hat{A}|+\rangle =|+\rangle$ and $\hat{A}|-\rangle =-|-\rangle$ . In the WVA technique, the system can be divided into three parts: pre-selection, coupling interaction, and post-selection. As shown in Eq. 7, the pre-selection part can be expressed as: |*ψ*_*i*_⟩ = |*r*_*p*_|cos*α*| + ⟩ + |*r*_*s*_|sin*α*| − ⟩. And $\hat{U}=e^{iK\left(\lambda ,n_{\text{d}}\right)\hat{A}}$ represents the unitary operator of the interaction between the system and the meter. The unitary operator $\hat{U}$ contains the system operator $\hat{A}$ and the wavelength operator *λ*. Therefore, the meter is chosen as the wavelength spectrum of light. For simplicity, we assume that the initial state |Φ⟩ = *∫f*(*λ*)|*λ*⟩d*λ* of the meter satisfies the Gaussian distribution with the mean value *λ*_0_ = 830 nm and the variance *σ*

$$f\left(\lambda \right)=\frac{1}{{\left(2\pi \sigma^{2}\right)}^{1/4}}e^{-\frac{{\left(\lambda -\lambda_{0}\right)}^{2}}{4\sigma^{2}}}.$$ (8)

In the WVA protocol, a post-selection procedure is required, which is completed by the polarizer P2 in Figure 2. The postselected state can be expressed as

$$\begin{array}{cc} |\psi_{f}\rangle & =cos\beta |p\rangle +sin\beta |s\rangle \\ & =\frac{1}{\sqrt{2}}\left(e^{-i\beta }|+\rangle +e^{i\beta }|-\rangle \right), \end{array}$$ (9)

where *β* is the angle between the polarizer’s polarization axis and horizontal direction.

Therefore, the spectral distribution *∫p*(*λ*)d*λ* after post-selection can be written as (without normalization)

$$p\left(\lambda ,n_{\text{d}}\right)=|\langle \psi_{f}|\hat{U}|\psi_{j}\rangle {|}^{2}\langle \Phi |\Phi \rangle .$$ (10)

And the relative reflectivity curve *R* (*λ*, *n*_d_) can be obtained

$$\begin{array}{c} R\left(\lambda ,n_{\text{d}}\right)=p\left(\lambda ,n_{\text{d}}\right)/\langle \Phi |\Phi \rangle . \end{array}$$ (11)

In this scheme, the values of *α* and *β* are chosen to satisfy: |*r*_*p*_|cos*α* = −|*r*_*s*_|sin*α* = *A*, (*k*_1_ − *l*)*λ*_0_ + *k*_2_ (*n*_d_) − *φ*_*s*_ + 2*β* = 0. Hence, we have

$$R\left(\lambda ,n_{\text{d}}\right)=2A^{2}{sin}^{2}\frac{\left(k_{1}-l\right)\lambda +k_{2}\left(n_{\text{d}}\right)-\varphi_{s}+2\beta }{2}.$$ (12)

Similar to conventional SPR sensors, there is an SPR dip in the reflectivity curve in this system, as shown in Figures 2C,D. When *l* = 0, the WMSPR scheme is equivalent to the conventional phase interrogation SPR. As shown in Figure 2C, the SPR dip’s position varies with the dielectric medium’s refractive index. However, the sensitivity of the WMSPR scheme can be controlled by modulating the value of *l*. In Figure 2D, we choose *l* = 3 × 10^−3^ rad/nm. The shift of SPR dip is now greater than conventional phase interrogation SPR.

**FIGURE 3:**
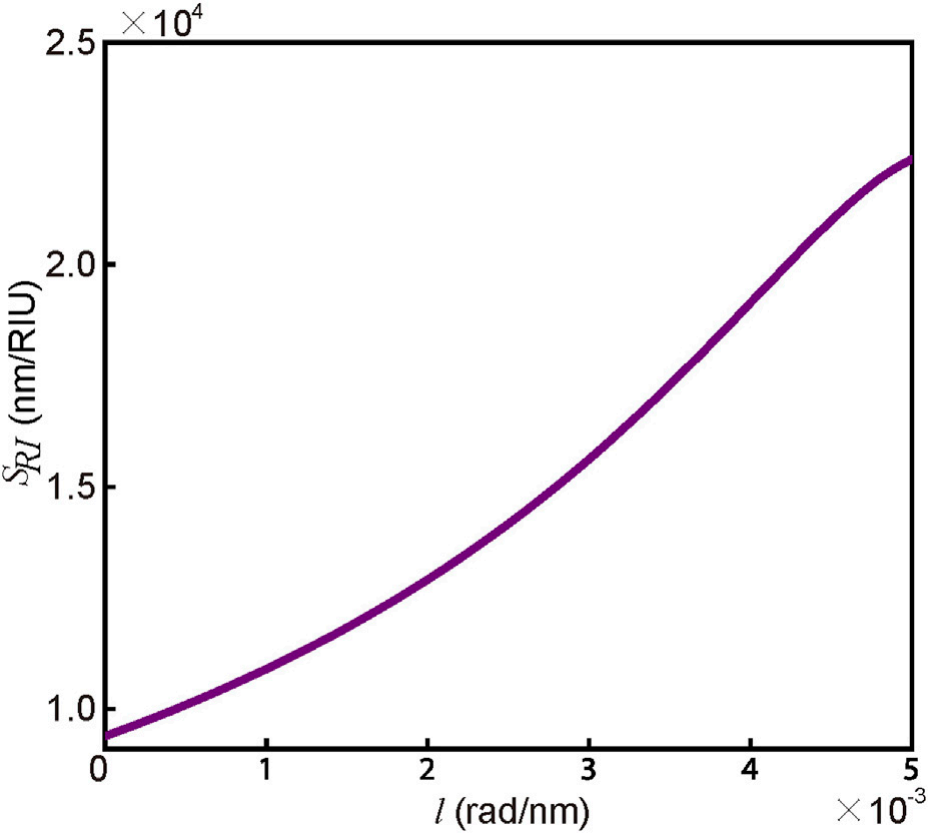
The relationship between the system’s sensitivity and before in the WMSPR scheme.

According to Eq. 12, the refractive index sensitivity *S*_*RI*_ of WMSPR can be calculated as

$$S_{RI}=\frac{1}{k_{1}-l}\frac{dk_{2}\left(n_{\text{d}}\right)}{dn_{\text{d}}},$$ (13)

which is amplified with a coefficient of 1/(*k*_1_ − *l*). We define the coupling strength as *τ* = *k*_1_ − *l*. Similar to rotatory biased weak measurement (Zhou et al., 2023), the system’s sensitivity is amplified by the factor of 1/*τ*. Therefore, the sensitivity of the WMSPR scheme can be enhanced by introducing an approximate value of *l*. Figure 3 depicts the relationship between the sensitivity *S*_*RI*_ of the system and *l*.

Figure 1 shows the structure of the WMSPR system, which consists of an SLD (IPSDD0805, *λ*_0_ = 830 nm, *σ*_0_ = 28 nm, 10 mW, Inphenix), an equilateral prism (made of ZF6 glass) and a spectrometer (Ocean Optics HR4000) to detect wavelength spectrum. The sensing surface of the prism is vaporized with a thin film of 5 nm chromium and 35 nm Au using an electron beam. The role of the cadmium film is to make the Au film more firmly attached to the surface of the prism. A 3D-printed resin chip is glued to the Au film using epoxy resin. The normal angle between the detecting light and the Au film is about 52°. To demonstrate the enhancement of sensitivity and resolution of the WMSPR system, this work performs experiments on both phase SPR system and WMSPR system.

## 3 Results and discussion

### 3.1 Calibration of the sensor system

Sodium chloride solution is usually considered a solution with a uniform refractive index and is used to measure the refractive index sensitivity and resolution of the optical sensing system. In the phase SPR sensors, deionized water is passed into the flow channel, and the front polarizer is adjusted to *π*/4, the rear polarizer is *ϵ* + 3*π*/4. By fine-tuning the quarter-wave plate and the rear polarizer, the spectrum of the detection point shows a double-peaked state, like the inset of Figure 5B. Subsequently, the syringe pump is used to pass different concentrations of sodium chloride solution. The correspondence between the concentration of sodium chloride solution (C, g/L) and its refractive index (*n*_*RI*_) is *n*_*RI*_ = 1.3305 + 1.47 × 10^−4^ ×C. We used the fixed boundary (the lower boundary is 780 nm, the upper boundary is 880 nm) spectrum to get the fitting curve of Eq. 11, from which we can get the shift of the SPR dip with the change of the refractive index. And the refractive index sensitivity and resolution can be obtained from *S*_*RI*_ = *δλ*/*δn* and *Res* = 3*σ*/*S*_*RI*_, *σ* is the standard deviation of the sensor.

In WMSPR, the adjustment of the coupling strength *τ* needs to be realized by SBC based on the phase SPR’s double-peak spectrum. After the adjustment, the phase SPR’s double-peak spectrum is destroyed. It is necessary to adjust the polarizers P1 and P2 until the spectrum appears double peaks, i.e., after adjusting the coupling strength, the system must reach the weak measurement state again. The exact calculation of sensitivity and resolution was also performed on the WMSPR.

**FIGURE 4:**
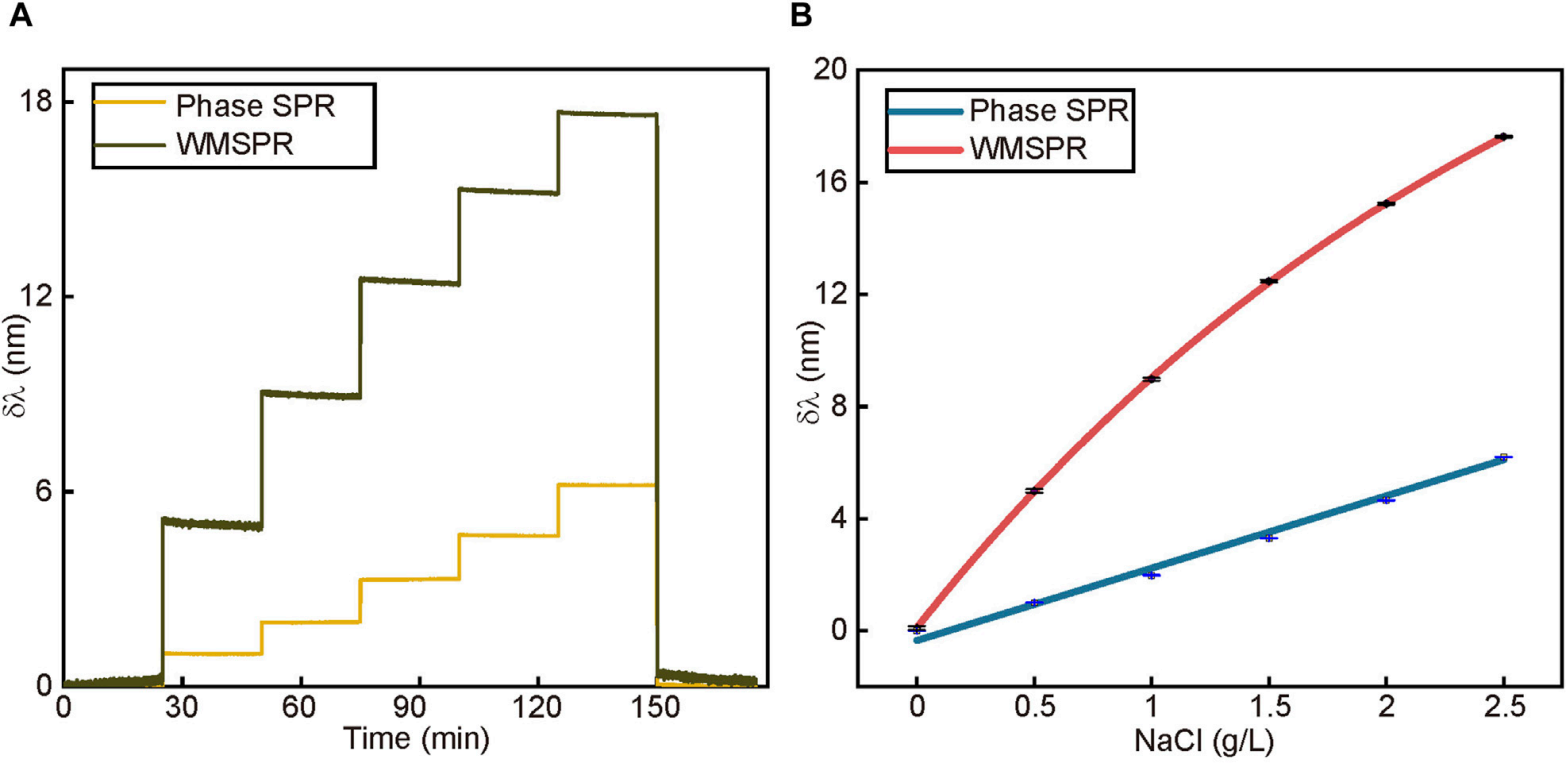
**(A)** The resonance wavelength change process caused by different concentrations of sodium chloride solution which are 0, 0.5, 1, 1.5, 2, 2.5 g/L from left step to right. The green line belongs to WMSPR and the yellow belongs to phase SPR. **(B)** The linear fitting curves of response to solution. Their slope is proportional to the sensor’s sensitivity. The red line and the blue line belong to WMSPR and phase SPR, respectively.

Sodium chloride solutions at 0.5, 1, 1.5, 2, 2.5, and 3 g/L were used for optical properties determination. In Figure 4A, the spectral offsets of the two systems are shown for sodium chloride solution with a gradient of concentration. As shown in Figure 4B, the refractive index sensitivity of phase SPR is 1.680 × 10^4^ nm/RIU, and the refractive index sensitivity of WMSPR is 4.737 × 10^4^ nm/RIU, which is about three times that of phase SPR. The calculated resolution is 6.333 × 10^−8^ RIU. The resolution of the sensor after adjusting the coupling strength is much better for the amount of refractive index change on the surface of the Au film. This means that we adjust the coupling strength to further amplify the signal while the amplification of the system noise is much lower than the amplified value of the signal.

### 3.2 Performance comparison of sensors

Interfacial sensor parameters usually are obtained using homogeneous standard solutions, which might is not suitable for measuring the performance in biological applications. This is because the biomolecular behavior cannot be regarded as a homogeneous process in the same way as replacing the sodium chloride solution. For general immunoassay analytes, specific behavior occurs within a few nanometers to tens of nanometers from the sensing surface. We will view the overall refractive index change within the penetration depth as the surface refractive index with respect to the sensing interface, often referred to as the bulk refractive index. Molecular interactions trigger a change in the bulk refractive index, which in turn triggers a change in the phase of the reflected photons. Our immune molecule-specific recognition experiments well validated the enhanced performance of WMSPR on molecular interactions.

**FIGURE 5:**
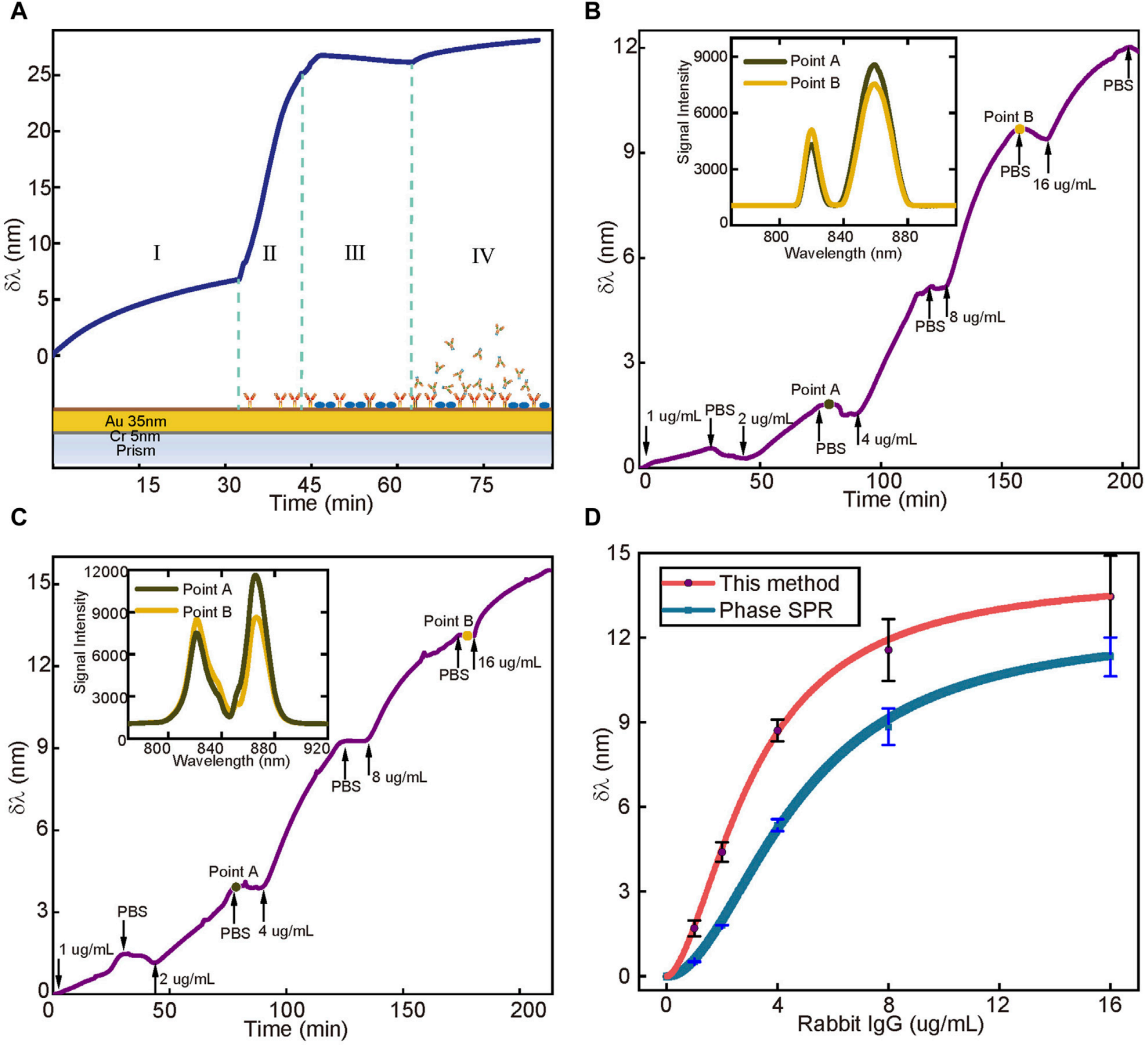
**(A)** The process of the functionalization of the WMSPR’s sensing surface, which happens in phase SPR as well. I is the process of self-polymerization of the Au film to form a polydopamine membrane. II is the process of the non-specific adsorption of goat anti-rabbit IgG on the polydopamine membrane. III is the process of blocking non-specific sites of the polydopamine membrane. IV is the process of specific binding between goat anti-rabbit IgG and rabbit IgG. **(B)** The real-time process of phase SPR sensor’s response to different concentrations of Rabbit IgG. The inset shows obvious variations of the spectra of point A and point **(B, C)** The real-time process of the WMSPR sensor’s response to different concentrations of Rabbit IgG. The inset shows more obvious variations than phase SPR’s **(D)** The total response value of each concentration of rabbit IgG, and the first three concentrations well demonstrate the enhancement sensitivity of WMSPR.

In the specific recognition experiment of antigen and antibody, we give a schematic diagram of the chip functionalization and the response signals in each process in Figure 5A. The first stage is the self-assembly of dopamine molecules into a polydopamine layer on the surface of the Au film. A very obvious change in the signal can be seen, followed by the adsorption of 10 μg/mL goat anti-rabbit IgG molecules to the polydopamine layer. In the third stage, the closure of the surface completes the chip functionalization. The last stage shows the specific binding stage. The inset of Figure 5B shows the spectra in both phase SPR and WMSPR sensing systems for the same detection point, which exhibits the spectral change from Point A to Point B, allowing obtaining the spectral offset. We can clearly see the change in the spectrum with time. This demonstrates the real-time, *in situ* detection capability of the WMSPR sensing system for molecular interactions. Figure 5B, (c) shows the total process of phase SPR and WMSPR for the specific recognition of goat anti-rabbit IgG and rabbit IgG. We recorded the amount of spectral change caused by 1, 2, 4, 8, and 16 μg/mL rabbit IgG captured by goat anti-rabbit IgG within 30 min using PBS as the starting baseline. Each specific binding procedure was followed by PBS for approximately 15 min to clear the channel and provide a new reference baseline. The fluctuation in the curve is due to the vibration caused by the replacement of the solution. In Figure 5D, the sensitivity of WMSPR was distinctly higher than that of phase SPR at low concentrations. The limit of detection of WMSPR and phase SPR can be calculated based on the 3*σ* principle of 5.301 ng/mL and 17.44 ng/mL, respectively. As such, it is clear that WMSPR has a superior detection limit. Since we limit the molecular interaction time to 30 min, the specific binding does not reach equilibrium, and the resulting calculated detection limit is larger than the actual performance. In practical application, if we wait until equilibrium, WMSPR could perform better than the detection limit given above.

## 4 Conclusion

In this work, a biosensor based on the weak value amplification technique and surface plasmon resonance is proposed. Based on the principle of the weak value amplification technique, an SBC is introduced to modulate the coupling strength of the system, and the sensitivity of the sensor is proportional to the inverse of the coupling strength. Our proposed scheme achieves a refractive index sensitivity of 4.737 × 10^4^ nm/RIU and a refractive index resolution of 6.333 × 10^−8^ RIU, which is three times higher than the conventional phase SPR. The sensor is also validated by IgG detection with a detection limit of 5.3 ng/mL. The results can be applied to the development of various highly sensitive plasmonic sensors.
