# Supplementary material for: An ultra-sensitive biosensor based on surface plasmon resonance and weak value amplification
Source: Front Chem. 2024 Mar 8;12:1382251. doi: 10.3389/fchem.2024.1382251 (PMC10957547; doi:10.3389/fchem.2024.1382251)
Supplement: Supplementary file 1 [file DataSheet1.pdf]

frontiers

Supplementary Material

1 THE SCIENCE OF WEAK VALUE AMPLIFICATION

Based on the von Neumann theory of measurement and the weak value amplification theory of AAV, we approximate the measured system state as:

$$\begin{aligned} |\Psi\rangle &= e^{-ig\hat{A}\hat{p}}|\psi\rangle|\Phi\rangle \\ &\simeq (1 - ig\hat{A}\hat{p})|\psi\rangle|\Phi\rangle \\ &= (1 - ig\langle\hat{A}\rangle\hat{p})|\psi\rangle|\Phi\rangle - ig\hat{p}\Delta\hat{A}|\psi^+\rangle|\Phi\rangle \end{aligned} \tag{S1}$$

$\langle\hat{A}\rangle = \langle\psi|\hat{A}|\psi\rangle, \Delta\hat{A} = \langle\psi|(\hat{A} - \langle\hat{A}\rangle)^2|\psi\rangle$ are the mean and the corresponding variance of $\hat{A}$ in the initial state of the system $|\psi\rangle$, respectively. $|\psi^+\rangle$ and $|\psi\rangle$ are perpendicular to each other.
The probability that the system remains in its initial state after the measurement is,

$$\frac{||(1 - i\langle g\hat{A}\rangle\hat{p})|\psi\rangle||}{||(1 - ig\hat{A}\hat{p})|\psi\rangle||} = \frac{1 - g^2\hat{q}^2\langle\hat{A}\rangle^2}{1 + g^2\hat{q}^2\langle\hat{A}^2\rangle} \sim 1 \tag{S2}$$

and the probability that the system is in $|\psi^+$ is,

$$\frac{2g^2\hat{q}^2\langle\hat{A}\rangle^2}{1 + g^2\hat{q}^2\langle\hat{A}^2\rangle} \sim 0 \tag{S3}$$

When the coupling strength g is small enough, the system state is almost unaltered. In this case, the probability distribution of the pointer probability distribution of the pointer state in spatial coordinates is,

$$p(q) = \sum_j |c_j|^2 \text{exp}\left[-\frac{(q - ga_j)^2}{2\sigma^2}\right] \tag{S4}$$

this distribution can be approximately as a Gaussian distribution,

$$p(q) \simeq \exp\left[-\frac{(q - g\langle\hat{A}\rangle)^2}{2\sigma^2}\right] \tag{S5}$$

Therefore, under weak interaction conditions, the probability of collapse of the state function of the quantum system is minimal, but the weak measurements still yield useful information about the system. At this point, since $g \ll 1$, the average displacement of the pointer is almost zero, which is much smaller than the spread of the state of the measuring instrument, and there will be a large error in reading the displacement of the measuring instrument, i.e., the information that can be obtained from a single measurement is much smaller than that of a strong measurement. Aharonov et al. found that the measurement results would be completely different with a single strong measurement of the coupled state after the weak measurement, projecting the quantum system to a state close to the initial one perpendicular to the post-selected state. When post-selection is successful, as the system discards the unperturbed state, the probability of post-selection will be very low. However, the displacement of the measuring instrument is amplified, a phenomenon known as weak value

amplification. As shown in Fig. S1,after interacting with the system and the pointer, the system is projected onto A by post-selection. The initial state of the system is assumed to be B. The coupled state after projection can be written,

$$\begin{aligned} |\Psi_{fin}\rangle &= |\psi_f\rangle\langle\psi_f|e^{-ig\hat{A}\hat{p}}|\psi_i\rangle|\Phi\rangle \\ &\simeq |\psi_f\rangle\langle\psi_f|(1-ig\hat{A}\hat{p})|\psi_i\rangle|\Phi\rangle \\ &= |\psi_f\rangle\langle\psi_f|\psi_i\rangle(1-igA_w\hat{p})|\Phi\rangle \\ &\simeq \langle\psi_f|\psi_i\rangle e^{-igA_w\hat{p}}|\psi_f\rangle|\Phi\rangle, |\mathbf{g}A_w| \ll 1 \end{aligned} \tag{S6}$$

A_w is the weak value, which is,

$$A_w = \frac{\langle\psi_f|\hat{A}|\psi_i\rangle}{\langle\psi_f|\psi_i\rangle} \tag{S7}$$

when the coupling strength is small enough to satisfy $|gA_w| \ll 1$, the detection probability is $p_f = |\langle\psi_f|\psi_i\rangle|^2$, the approximate distribution of wavefunction is $p_f\phi(q - g\mathrm{Re}A_w)$. The probability of detection for weak measurements is reduced to $p_f \ll 1$, but the displacement of the pointer is multiplied by a factor $\mathrm{Re}A_w$. When $\mathrm{Re}A_w$, ehe amplification of the pointer displacement due to the coupling between the instrument and the system is the weak value amplification effect proposed by AAV et al.

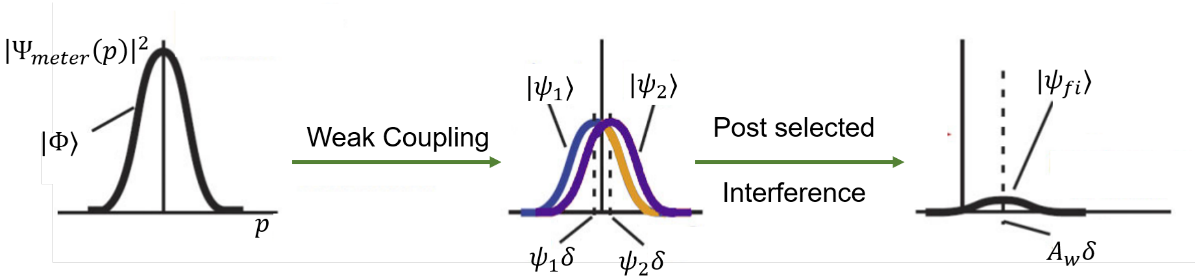

Figure S1. Weak Measurement Model.

In the standard weak measurement scheme, the frequency domain centre of gravity momentum shift of the photon after the weak measurement is,

$$\delta P = 2k(\Delta P)^2\mathrm{Im}A_w \tag{S8}$$

in this equation, ΔP is the variance of the light source spectrum, $A_w \simeq \frac{1}{\beta - i\alpha}$, $\mathrm{Im}A_w = \alpha/(\beta^2+\alpha^2)$, β is the offset angle in the orthogonal direction between the post-selected state and the pre-selected state, α is the measured phase, light source's central wavelength is λ_0, the momentum is P_0, coupling strength $k = \frac{\alpha}{P_0}$ is extremely small, then there's

$$\delta P = \frac{2\Delta P^2\alpha^2}{P_0(\beta^2+\alpha^2)} \tag{S9}$$

Meanwhile, we can observe the changes in centre wavelength,

$$\delta\lambda = \frac{2(\Delta\lambda)^2\alpha^2}{\lambda_0(\beta^2+\alpha^2)} \tag{S10}$$

In a word, coupling constant g, and pre, post-selected states need to satisfy

$$g\Delta p \ll \frac{|\langle\psi_f|\psi_i\rangle|}{|\langle\psi_f|\hat{A}^n|\psi_i\rangle|^{1/n}} \quad \text{for } n = 1, 2, \cdots \tag{S11}$$

but this condition can't be satisfied when the pre- and post-selected states are orthogonal. Researchers found that the amplification effect remains, and called it the inverse linear region (Wu and Li, 2011; Lyons et al., 2018). In this scheme, the pre- and post-selected states are orthogonal, which means $\langle\psi_f|\psi_i\rangle = 0$. Standard weak measurement don't apply in this case. So we calculated the probability density distribution function in the frequency domain after post-selection.

$$\begin{aligned} p(P) &= \int \mathrm{d}P |\langle P|\langle\psi_f|e^{-ik\hat{A}\hat{p}}|\psi_i\rangle|\Phi\rangle|^2 \\ &= \frac{1}{[2\pi(\Delta P)^2]^{1/2}} \sin^2(kP)\mathrm{cos}p\left[-\frac{(P-P_0)^2}{2(\Delta P)^2}\right] \end{aligned} \tag{S12}$$

At the same time, because the pre- and post-selected states are orthogonal in the inverse linear region, there's a zero point P_1 in the distribution of the probability density distribution, $\sin(kP_1) = 0$. And the measurement probability and the centre of gravity momentum displacement results

$$\begin{aligned} p_f &= \int \mathrm{d}P \frac{1}{[2\pi(\Delta P)^2]^{1/2}} \sin^2(kP)\mathrm{exp}\left[-\frac{(P-P_0)^2}{2(\Delta P)^2}\right] \\ &\simeq \frac{1-e^{-2k^2\Delta P^2 \cos(kP_0)}}{2} \\ \delta P &= \frac{1}{p_f}\int \mathrm{d}P P p(P) \\ &= \frac{2k\Delta P^2(kP_0 - m\pi)}{k^2\Delta P^2 + 2(kP_0 - m\pi)^2} \\ &\sim \frac{2(kP_0 - m\pi)}{k} \end{aligned} \tag{S13}$$

in this equation, m is integer, and $kP_1 - m\pi = 0$. The gain coefficient of the inverse linear region on the displacement of the centre of gravity momentum is $k_1 = m\pi/k^2$. Compared to the Eq. S8, standard weak measurement's gain foefficient is $k_2 = 2(\Delta P)^2 \mathrm{Im} A_w$, its constraint is

$$k\Delta p \ll \frac{1}{\mathrm{Im}A_w} \ll 1 \tag{S14}$$

Weak measurement system used in this work has

$$2(k\Delta p)^2\mathrm{Im}A_w K m\pi$$
$$k_1 = \frac{m\pi}{k^2} \gg 2(\Delta P)^2\mathrm{Im}A_w = k_2 \tag{S15}$$

And we can achieve higher gain factor, meanwhile the smaller the coupling factor k, the greater the gain factor k_1.

2 CALCULATION OF REFLECTION COEFFICIENT

The residual iterative formulations of Eq. (2) in the manuscript are as follows.

$$r_{25}^{p(s)}(\lambda) = \frac{r_{23}^{p(s)}(\lambda) + r_{35}^{p(s)}(\lambda)\exp(2ik_{3z}(\lambda)d_3)}{1 + r_{23}^{p(s)}(\lambda)r_{35}^{p(s)}(\lambda)\exp(2ik_{3z}(\lambda)d_3)} \tag{S16}$$

$$r_{35}^{p(s)}(\lambda) = \frac{r_{34}^{p(s)}(\lambda) + r_{45}^{p(s)}(\lambda)\exp(2ik_{4z}(\lambda)d_4)}{1 + r_{34}^{p(s)}(\lambda)r_{45}^{p(s)}(\lambda)\exp(2ik_{4z}(\lambda)d_4)} \tag{S17}$$

In these formulas, we label the prism, chromium layer, gold layer, polydopaminelayer and dielectric medium with 1, 2, 3, 4 and 5. d_j denotes the thickness of layer j and k_{jz} denotes the z-direction wave vector of layer j. Meanwhile,

$$k_{jz} = \sqrt{\epsilon_j(\omega/c)^2 - k_x^2}, \tag{S18}$$

$$k_x = \sqrt{\epsilon_1(\omega/c)^2}\sin\theta, \epsilon_1 = n_p^2 \tag{S19}$$

$$X_j^p = \frac{\epsilon_j}{k_{jz}}, X_j^s = k_{jz}, j = 1, 2, 3, 4, 5 \tag{S20}$$

$$r_{j-1,j} = \frac{X_{j-1}^p - X_j^p}{X_{j-1}^p + X_j^p}, j = 2, 3, 4, 5 \tag{S21}$$

3 CALCULATION MODEL SUPPLEMENT

First, for the consideration of the thickness of the chromium layer, we first use the Drude-Lorentz model to calculate the complex permittivity of the chromium layer with respect to the wavelength. Through the surface plasmon resonance conditions, no surface plasmon resonance occurs at the chromium-prism interface, so we can just treat chromium as a waveguide. And the chromium layer can still be added to the multilayer model for calculation. Thus we give the sensitivity following the modulation strength by varying the thickness of the chromium layer. As shown in Figure S2, we give conditions that are as consistent as possible with the actual situation, e.g. a 35 nm gold film and an equivalent refractive index model containing a polydopamine layer. It is clear that a change in the thickness of the chromium layer will only cause a flattening of the overall magnitude of the sensitivity curve, which does not prevent the adjustable sensitivity of the desired response. In the vapour deposition process, a thin layer of chromium is often used to allow better adhesion of the gold film to the glass, so we have omitted consideration of cadmium in the manuscript.

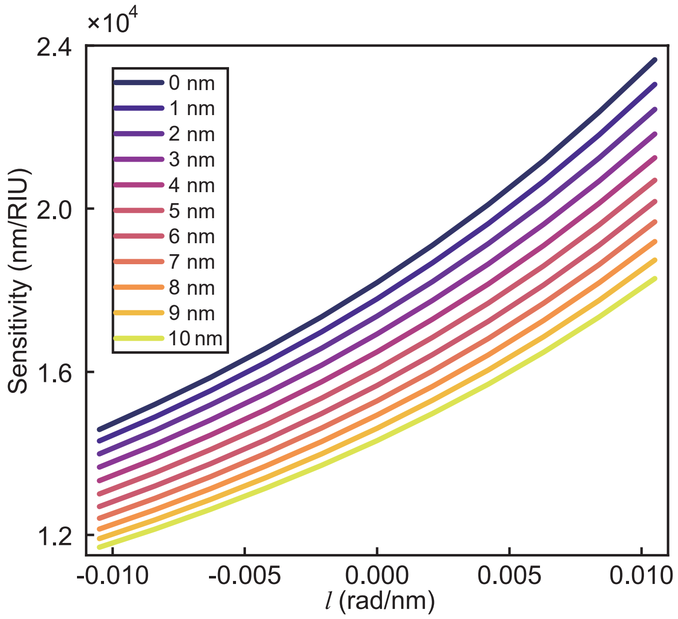

Figure S2. Sensitivities to different thickness of Cr.

Secondly, we first simulated two scenarios separately. One was the central wavelength shift when the equivalent refractive index of the medium layer in contact with the gold film changed, without considering the biomolecular film, as shown in Figure S3.a. The other was the central wavelength shift caused by different polydopamine film thicknesses, as shown in Figure S3.b. We correlated these two based on the correspondence between central wavelength shifts, and obtained the relationship between the equivalent polydopamine film thickness and equivalent refractive index through fitting, as shown in Figure S3.c. Finally, we present the relationship between equivalent refractive index and system sensitivity. We can see that changes in the polydopamine film thickness also lead to changes in sensitivity, shown in Figure S3.d. However, combined with Figure S3.c, the change diminishes and stabilizes eventually, which is determined by the characteristics of the evanescent wave. Therefore, the different polydopamine film thicknesses do not affect the expression of the core method in this paper.

4 TEM AND EDS OF THE CROSS-SECTION

We first prepared samples for TEM observation by focused ion beam cutting (FIB). We then opted to use a transmission electron microscope (TEM) with higher resolving power, the FEI Tecnai G2 F30 model, to observe the film cross-section. In Fig. S4, we can see that the chromium layer thickness is approximately 9.7 nm, while the gold film thickness is around 32.2 nm. This discrepancy with the simulation conditions is likely due to limitations in the precision of the coating equipment. As noted in the response to another Reviewer's comment 10, variations in the chromium layer thickness would only shift the modulation intensity-sensitivity curves up or down, and should not impede conveying the core aspect of this work.
Fig. S6 also provides the Energy Dispersive Spectroscopy (EDS) results of the cross-section obtained

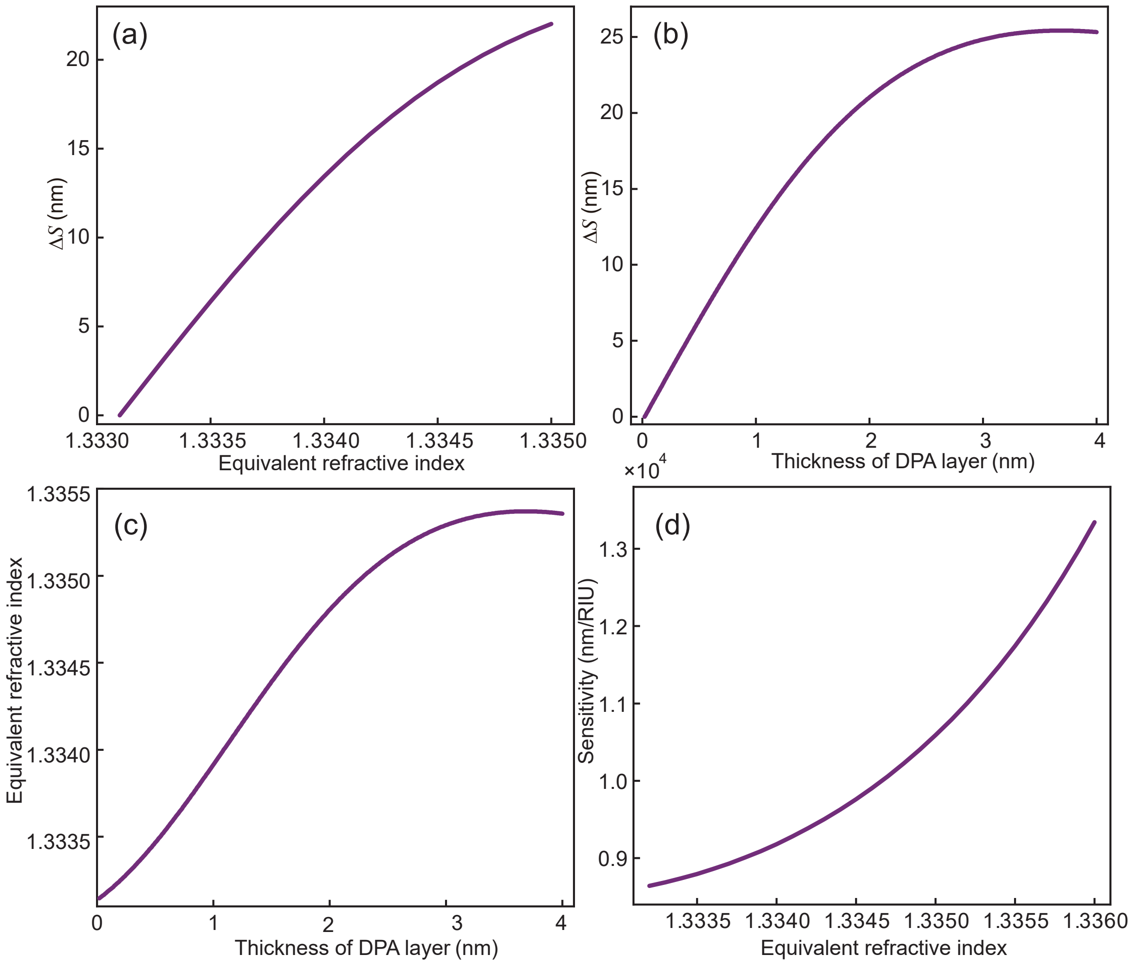

Figure S3. (a) Central wavelength shift induced by equivalent refractive index. (b) Central wavelength shift induced by varying polydopamine film thicknesses. (c) Relationship between equivalent refractive index and equivalent polydopamine film thickness. (d) Sensitivity change at different equivalent refractive indices.

via transmission electron microscopy, indicating the ratio of Au to Cr content.

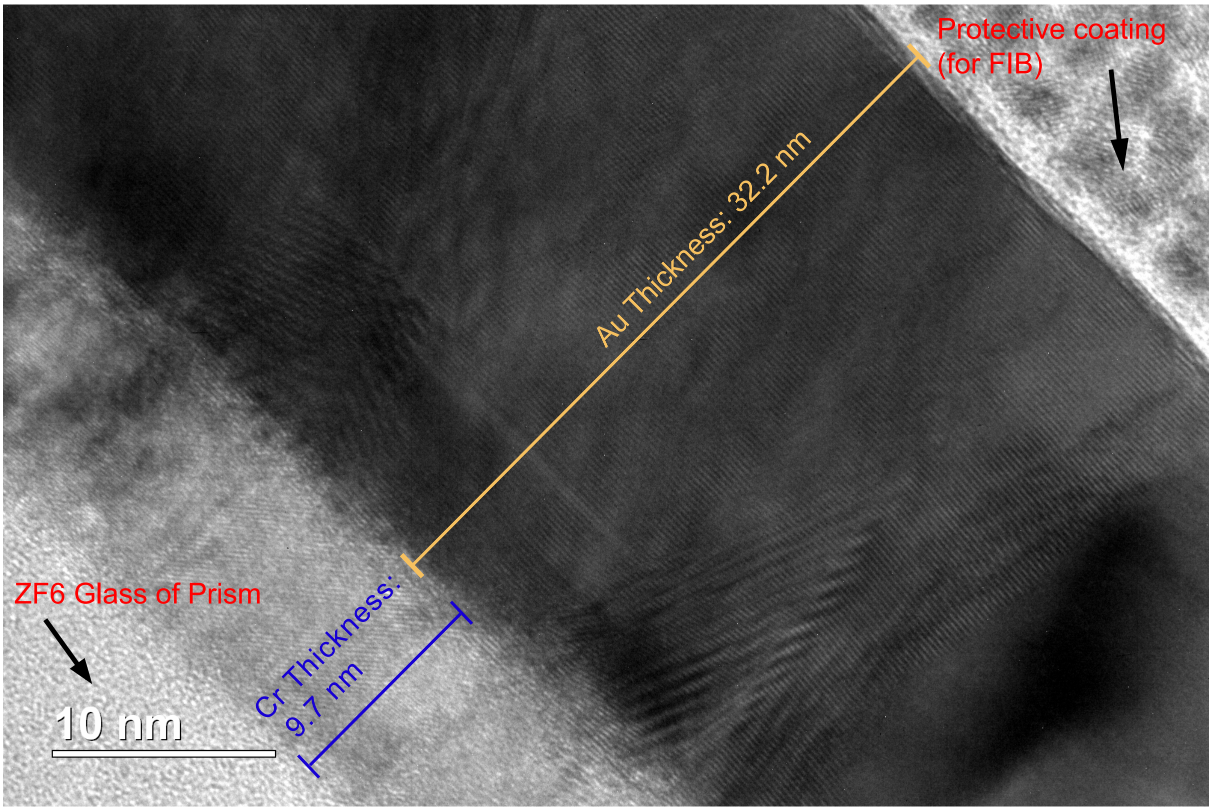

Figure S4. Detail TEM image of the cross section.

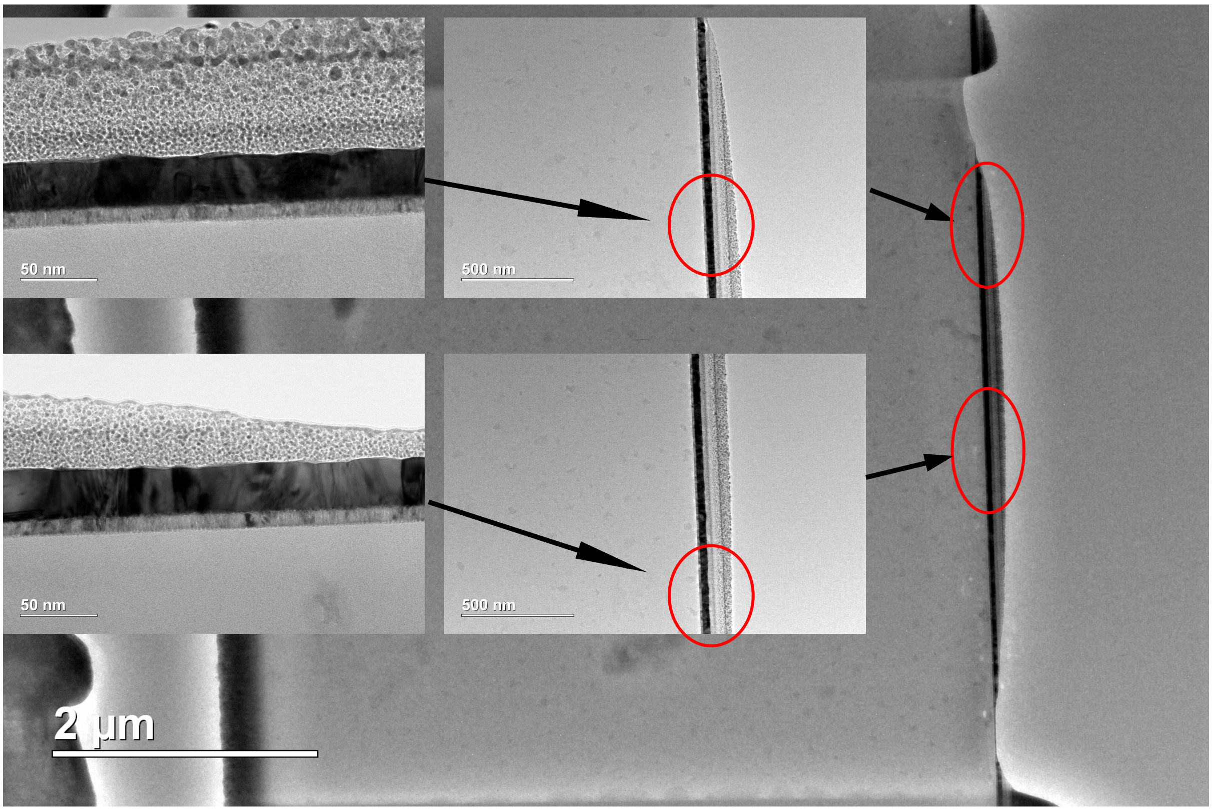

Figure S5. TEM image of the coating section after FIB sampling.

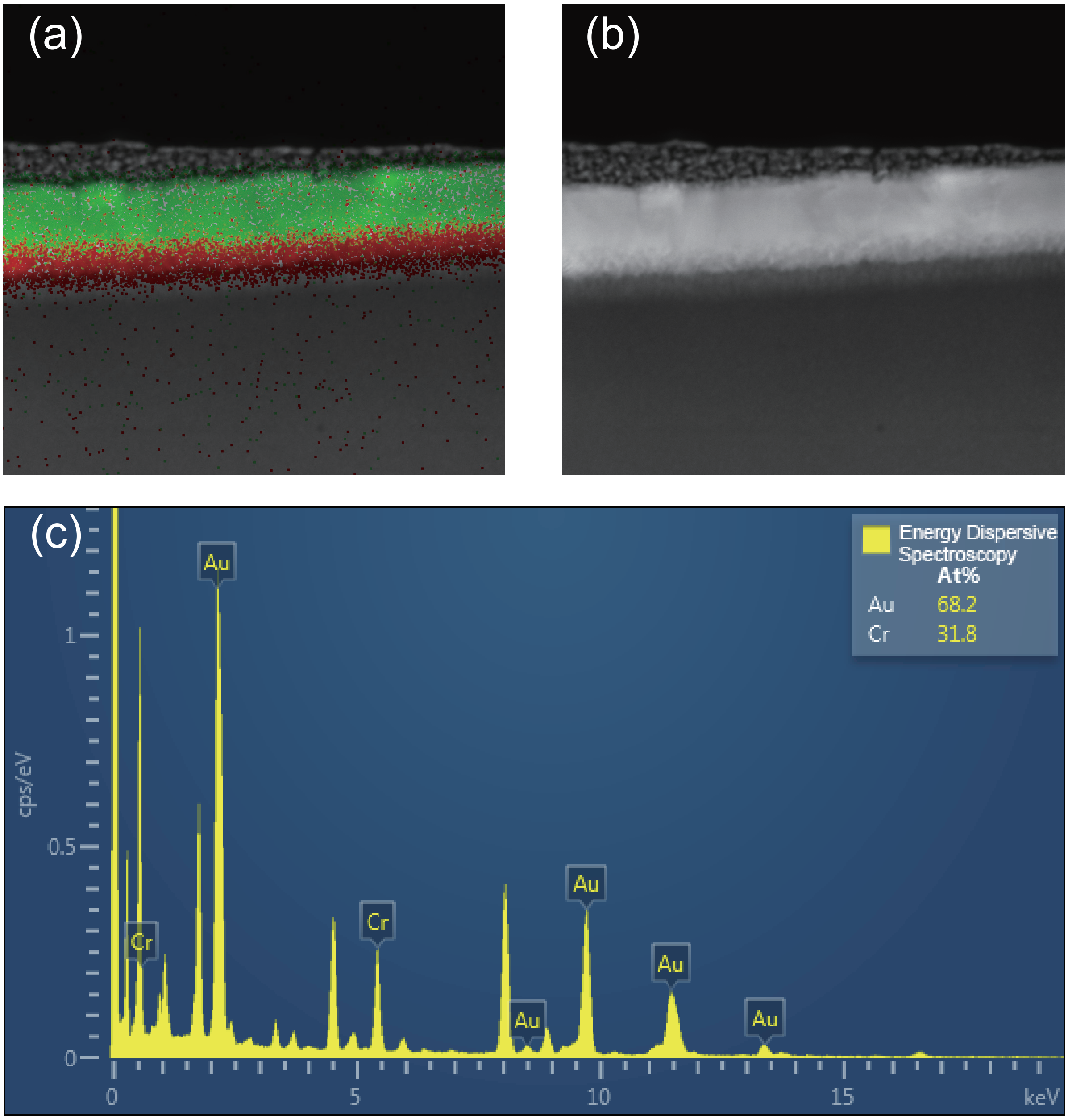

Figure S6. (a). Distribution of gold and chromium elements in the field of view. (b). TEM image of the view. (c). Energy dispersive spectroscopy of Au and Cr of the sample.

5 DYNAMIC RANGE

As shown in Fig .S7, 2.5 g/L concentration of the right peak of the curve just reached the upper limit of the measurement of the spectrometer used in our experiments, if the concentration continues to increase, the right peak will be overexposed beyond the range, and the double-peak equilibrium of the curve represents the concentration of 1 g/L, then we get half the dynamic interval of NaCl 1-2.5 g/L, i.e., the refractive index of 1.3347-1.3374 RIU. If we were to overexpose from the left peak to the right peak, then it would be the dynamic range, 0-0.0054 RIU. Meanwhile, we found from the response curve fit plots that the sensitivity enhancement of the scheme is still valid within this instrument-limited dynamic range interval.

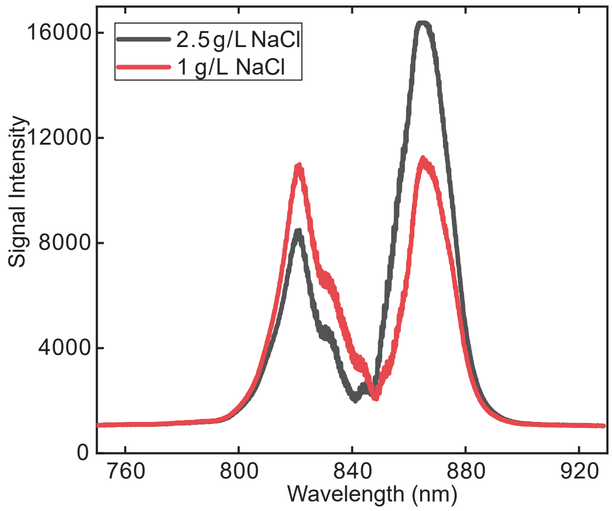

Figure S7. The spectrograms of the signals when the NaCl concentrations are 1 g/L and 2.5 g/L, respectively.

REFERENCES

Lyons, K., Howell, J. C., and Jordan, A. N. (2018). Noise suppression in inverse weak value-based phase detection. Quantum Studies: Mathematics and Foundations 5, 579–588

Wu, S. and Li, Y. (2011). Weak measurements beyond the aharonov-albert-vaidman formalism. Physical Review A 83, 052106
